# Supplementary material for: Interfacial water confers transcription factors with dinucleotide specificity
Source: Nat Struct Mol Biol. 2025 Jan 3;32(4):650–61. doi: 10.1038/s41594-024-01449-6 (PMC11996681; doi:10.1038/s41594-024-01449-6)
Supplement: Supplementary file 1 — Reporting Summary [file 41594_2024_1449_MOESM1_ESM.pdf]

nature portfolio

Corresponding author(s): NSMB-A48351-T, Jussi Taipale

Last updated by author(s): Oct 2, 2024

Reporting Summary

Nature Portfolio wishes to improve the reproducibility of the work that we publish. This form provides structure for consistency and transparency in reporting. For further information on Nature Portfolio policies, see our Editorial Policies and the Editorial Policy Checklist.

Statistics

For all statistical analyses, confirm that the following items are present in the figure legend, table legend, main text, or Methods section.

n/a	Confirmed	
☐	☒	The exact sample size (*n*) for each experimental group/condition, given as a discrete number and unit of measurement
☐	☒	A statement on whether measurements were taken from distinct samples or whether the same sample was measured repeatedly
☒	☐	The statistical test(s) used AND whether they are one- or two-sided<br>*Only common tests should be described solely by name; describe more complex techniques in the Methods section.*
☒	☐	A description of all covariates tested
☒	☐	A description of any assumptions or corrections, such as tests of normality and adjustment for multiple comparisons
☐	☒	A full description of the statistical parameters including central tendency (e.g. means) or other basic estimates (e.g. regression coefficient) AND variation (e.g. standard deviation) or associated estimates of uncertainty (e.g. confidence intervals)
☒	☐	For null hypothesis testing, the test statistic (e.g. *F*, *t*, *r*) with confidence intervals, effect sizes, degrees of freedom and *P* value noted<br>*Give P values as exact values whenever suitable.*
☒	☐	For Bayesian analysis, information on the choice of priors and Markov chain Monte Carlo settings
☒	☐	For hierarchical and complex designs, identification of the appropriate level for tests and full reporting of outcomes
☒	☐	Estimates of effect sizes (e.g. Cohen's *d*, Pearson's *r*), indicating how they were calculated

Our web collection on statistics for biologists contains articles on many of the points above.

Software and code

Policy information about availability of computer code

Data collection	Crystallographic data were collected using the software developed in synchrotron beam-line ID23-1 in ESRF and listed in Material and Method section
Data analysis	Crystallographic data analysis: XDS and CCP4 suits 7.1 and 8.0; MR and refinement: Phaser and Refmac5 as implemented in CCP4 and Phenix.refine; Model building: Coot (versions 0.9.6 and o.9.8.92 (EL)) as implemented in CCP4 and Phenix; Structural visualisation: PyMol 2.5.4; HT-SELEX data analysis: spacek40 (http://github.com/jttoivon/moder2/blob/master/myspacek40.c); Molecular Dynamic simulations in crystal lattice and analysis: AMBER21, UCSF Chimera, Amber 14SB force field and BSC1 parameters for DNA, TIP3P and Joung-Cheatham for water and ions parameters, pmemd.cuda, cpptraj tool, MDTraj, MAtplotlib; Multiple Sequence alignment: Clustal Omega (https://www.ebi.ac.uk/jdispatcher/); Molecular Dynamic simulations for analysis of entropy: GROMACS 2019 simulation package with LINCS algorithm implemented into GROMACS 2019, AMBER-19SB forcefield with OL15 modified parameters for DNA, Per|Mut for calculations of spatially resolved solvent entropies (https://gitlab.gwdg.de/lheinz/hydration_entropy), Python 3.11.8 and modules: NumPy 1.26.4, MDAnalysis 2.7.0, Matplotlib 3.8.4, and Pandas 2.2.2

For manuscripts utilizing custom algorithms or software that are central to the research but not yet described in published literature, software must be made available to editors and reviewers. We strongly encourage code deposition in a community repository (e.g. GitHub). See the Nature Portfolio guidelines for submitting code & software for further information.

Data

Policy information about availability of data

All manuscripts must include a data availability statement. This statement should provide the following information, where applicable:

- Accession codes, unique identifiers, or web links for publicly available datasets
- A description of any restrictions on data availability
- For clinical datasets or third party data, please ensure that the statement adheres to our policy

Crystal structures were deposited to the protein data bank (PDB) with accession codes: 7Z5I and 7Z5K for crystal structures of MYF5. The PDB code 1MDY was used for the Molecular replacement. The accession codes 8PMF, 8PMN, 8PMC, 8PM5, 8PM7, 8PMV, 8PN4, 8PNA and 8PNC are for BARHL2 structures. The accession code 3A01 was used for structure determination. The details are presented in Table 1. All sequence reads are deposited to the European Nucleotide Archive under the accession number PRJEB65950. The DNA ligands used in the Temperature HT-SELEX experiments are presented in Extended Data Table 3. The details are described in the Material& Method section.

Research involving human participants, their data, or biological material

Policy information about studies with human participants or human data. See also policy information about sex, gender (identity/presentation), and sexual orientation and race, ethnicity and racism.

Reporting on sex and gender	This study did not involve human participants
Reporting on race, ethnicity, or other socially relevant groupings	This study did not involve human participants
Population characteristics	This study did not involve human participants
Recruitment	This study did not involve human participants
Ethics oversight	Ethics permissions were not applicable to this study

Note that full information on the approval of the study protocol must also be provided in the manuscript.

Field-specific reporting

Please select the one below that is the best fit for your research. If you are not sure, read the appropriate sections before making your selection.

☒ Life sciences ☐ Behavioural & social sciences ☐ Ecological, evolutionary & environmental sciences

For a reference copy of the document with all sections, see nature.com/documents/nr-reporting-summary-flat.pdf

Life sciences study design

All studies must disclose on these points even when the disclosure is negative.

Sample size	Our study did not perform a formal sample size calculation. For solving crystal structures: a few dozens crystallization conditions were tested and at least 50 crystals of each complex were tested at the synchrotron beam-line to find the best diffraction. The data sets were collected from one crystal of each complex. For mutational analysis 14 single and double mutants were designed, synthesized and added to the HT-SELEX experiments.
Data exclusions	No data were excluded from the analysis
Replication	The X-ray data were collected from a single crystals of each complex. The structure of BARHL2/DNAAC was solved at two different resolution to be sure that the 0.95 Å resolution structure is the same as the structure at 1.3 Å resolution. The statistics of data collections and refinements are presented in Table 1. The DNA motif data were obtained from the multiple cycles of SELEX experiments. A total of four SELEX cycles were performed. The DNA ligands from 0, 3rd, and 4th SELEX cycles were sequenced. In the experiments with Temperature SELEX the ligands from each cycle of four cycles and the input were sequenced and analyzed. The conclusions drawn were supported by all SELEX cycles and/or replicates.
Randomization	No grouping was involved in the experiments - no randomization was conducted as a result.
Blinding	There were no groups or human or animal participants involved in the experiments - no blinding was required

Reporting for specific materials, systems and methods

We require information from authors about some types of materials, experimental systems and methods used in many studies. Here, indicate whether each material, system or method listed is relevant to your study. If you are not sure if a list item applies to your research, read the appropriate section before selecting a response.

Materials & experimental systems

n/a	Involved in the study
☒	☐ Antibodies
☒	☐ Eukaryotic cell lines
☒	☐ Palaeontology and archaeology
☒	☐ Animals and other organisms
☒	☐ Clinical data
☒	☐ Dual use research of concern
☒	☐ Plants

Methods

n/a	Involved in the study
☒	☐ ChIP-seq
☒	☐ Flow cytometry
☒	☐ MRI-based neuroimaging

Plants

Seed stocks	no
Novel plant genotypes	no
Authentication	no
